# The efficacy of Ahmed glaucoma valve drainage devices in cases of adult refractive glaucoma in Indian eyes

**DOI:** 10.4103/0301-4738.62667

**Published:** 2010

**Authors:** Ariga Murali

**Affiliations:** Swamy Eye Clinic, 11 South Mada Street, Villivakkam, Chennai-600 049, India

Dear Editor

**Figure 1:**
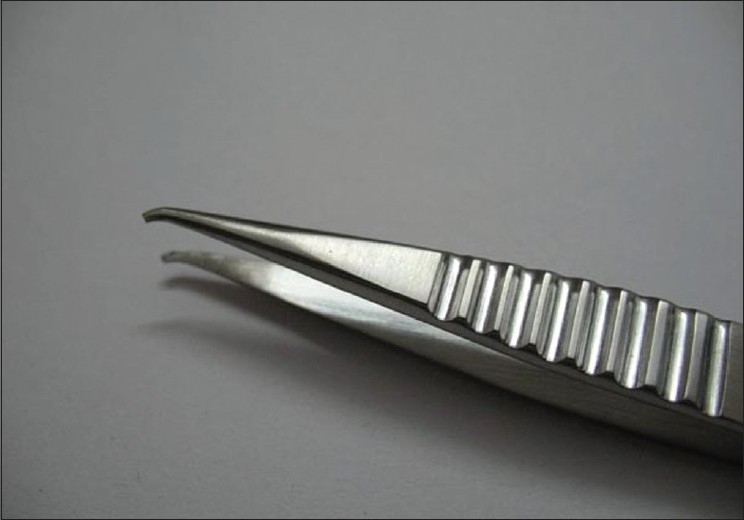
Tube insertion forceps

We read with interest the article by Parihar *et al*.[1] We congratulate the authors for their study and wish to make a few observations. While the authors have mentioned that tube insertion into the anterior chamber (AC) is done through a paracentesis track made with a 22-G needle and also that the use of viscoelastic through this needle track assists tube insertion there is no mention of the use of a special tube inserter forceps which we find greatly facilitates insertion of the Ahmed glaucoma valve (AGV) tube in to the AC. The tube inserter (New World Meditec Inc, CA) is a stainless steel forceps with a serrated grip and notched tip [Fig. 1] which provides rigidity to the AGV tube for easy insertion into the anterior or posterior chamber unlike any other serrated or tying forceps which may kink or damage the tube.

In situations where there is extensive conjunctival scarring and in revision surgeries the use of preserved scleral allograft obtained from an eye bank is suitable for covering the tube. It is biocompatible and can be trimmed to any size or shape. Ethanol-preserved sclera should be soaked in balanced salt solution for about 20 min before use. The use of such allografts has not been discussed by the authors.

The authors have enlisted many complications noted in their study following the use of the AGV in refractive glaucoma. Tube retraction is another complication which has been encountered by us in some cases. Tube retraction [Fig. 2a] refers to the retraction of the tube from the AC either into the scleral tunnel which had been fashioned for tube placement or into the subconjunctival space. This results in failure of aqueous drainage and resultant elevation in IOP. When this occurs and the tube is too short to be re-inserted the options for management include replacement with another AGV, reinsertion of the tube into pars plana (a pars plana clip may be used) or a tube extender[2] can be used to lengthen the tube. The tube extender (New World Meditec Inc, CA) is made of medical grade silicone with a length of about 24 mm and outer diameter of 0.635 mm. The proximal end can be attached to the existing AGV tube after removing it from the AC while the distal end can be inserted into the AC through a new paracentesis opening made with a 22-G needle. The tube extender is then sutured to the underlying sclera. We have used a tube extender in such situations [Figs. 2b and 2c] with good results.

**Figure 2a:**
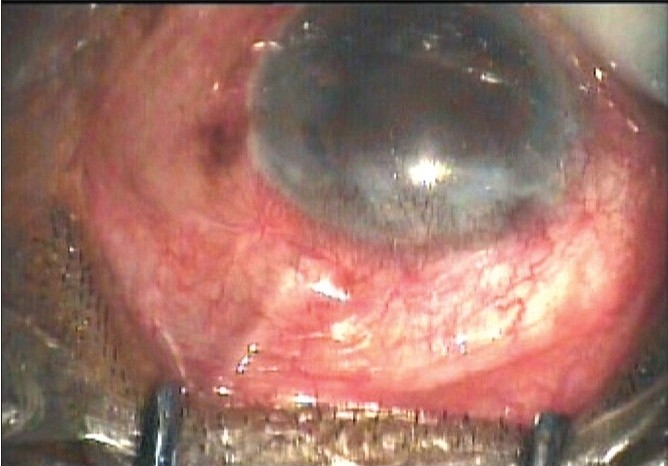
Tube retraction following AGV

**Figure 2b:**
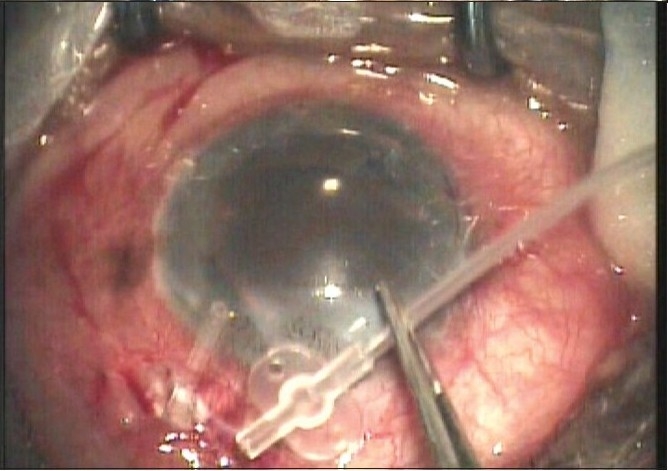
Tube extender

**Figure 2c:**
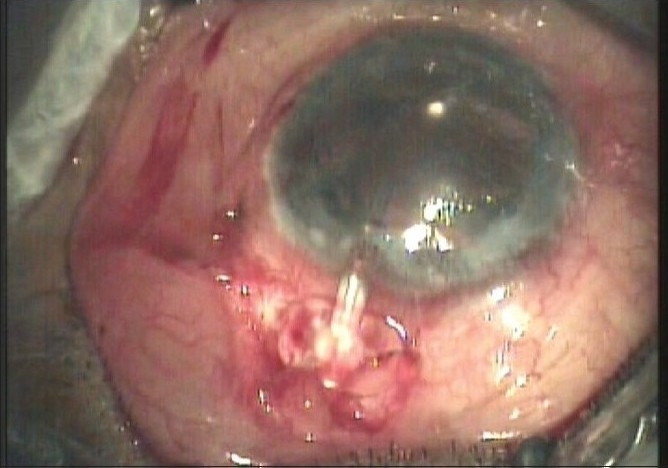
Tube extender inserted into the AC and sutured
